# Supplementary material for: Coverage bias in small molecule machine learning
Source: Nat Commun. 2025 Jan 9;16:554. doi: 10.1038/s41467-024-55462-w (PMC11718084; doi:10.1038/s41467-024-55462-w)
Supplement: Supplementary file 1 — Supplementary Information [file 41467_2024_55462_MOESM1_ESM.pdf]

Coverage bias in small molecule machine learning
Supplementary material

Fleming Kretschmer[1], Jan Seipp[2], Marcus Ludwig[1,3], Gunnar W. Klau[2], and Sebastian Böcker[1]

[1] Chair for Bioinformatics, Institute for Computer Science, Friedrich Schiller University Jena, Jena, Germany
[2] Algorithmic Bioinformatics, Institute for Computer Science, Heinrich Heine University Düsseldorf, Germany
[3] Currently at Bright Giant, Jena, Germany

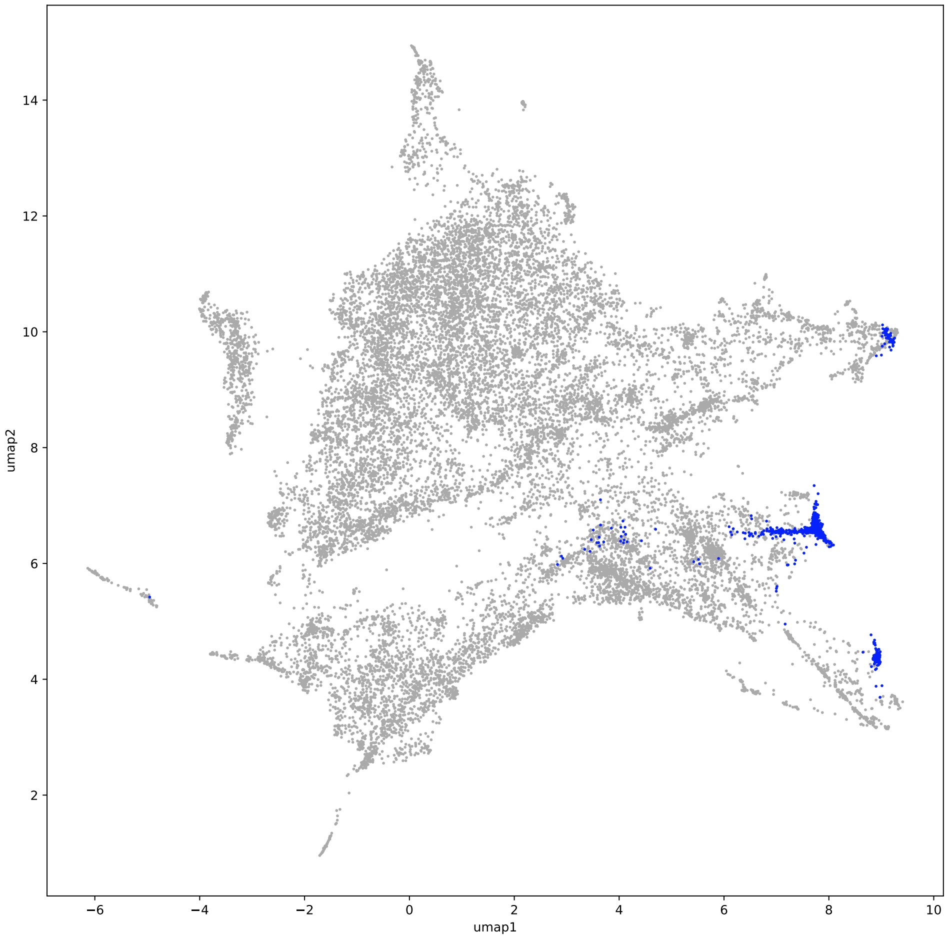

Supplementary Fig. 1. Full map of biomolecular structures. We did not recompute the UMAP embedding for this figure. Rather, the same UMAP embedding as in Fig. 3 was used. UMAP embedding computed from the 18,096 molecular structures. Shown in this plot are all 19,994 molecular structures subsampled from the biomolecular structures. Highlighted are the 1898 molecular structures not shown in Fig. 3. Compare to Fig. 1.

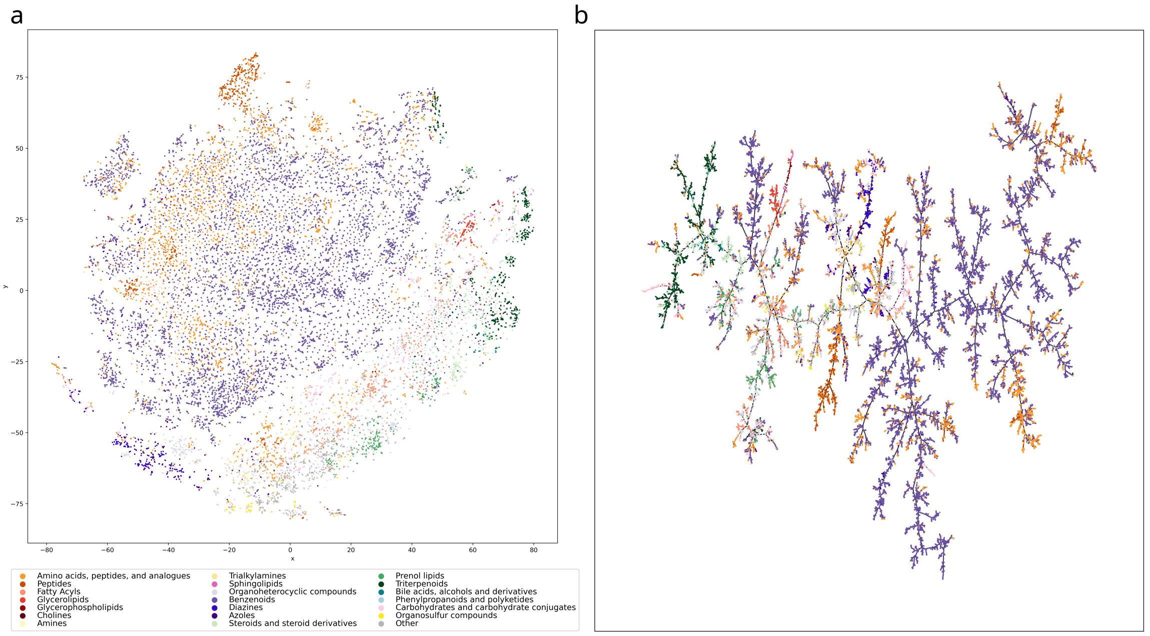

Supplementary Fig. 2. t-SNE and Minimum Spanning Tree visualizations. As alternative visualization methods to UMAP, t-SNE (a) and Minimum Spanning Tree computation (b) are applied to the pairwise myopic MCES distances of the 18,096 biomolecular structures (outlier lipid clusters excluded). The color-coding of compound classes from Fig. 1 is used. t-SNE computations are performed with scikit-learn[37], using the default settings (version 1.1.3). The Minimum Spanning Tree is computed using SciPy[38] version 1.10.1, based on Kruskal's algorithm[39].

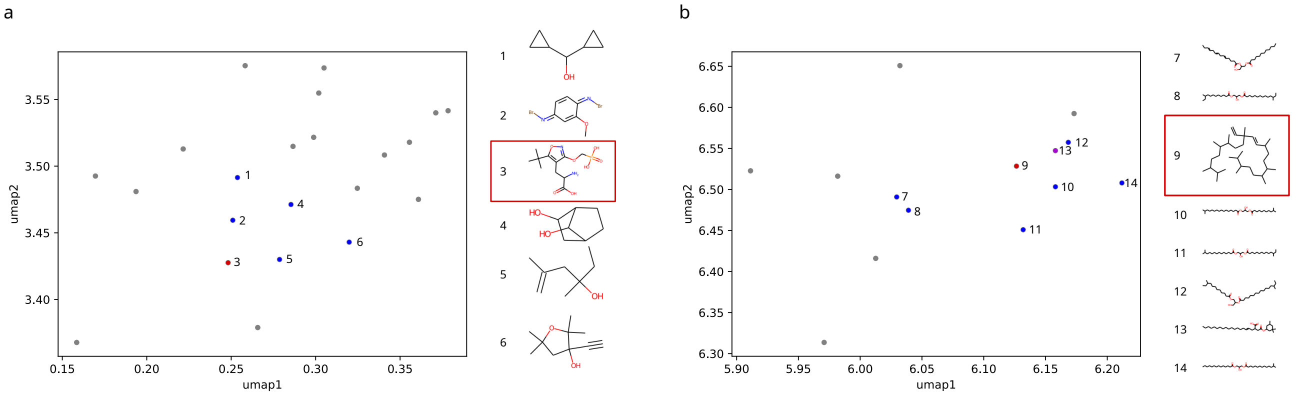

Supplementary Fig. 3. UMAP embeddings are clearly far from perfect. Both examples show a section of the MCES-based UMAP embedding of biomolecular structures (compare to Fig. 3). (a) This section of the embedding contains structures that do not share much similarity with one another, but with seemingly no better possible placement by UMAP. For instance, the structure labeled "3" is a member of the SMRT dataset mapped onto the UMAP space, with large myopic MCES distances (from 17.5 to 20.5) to all other labeled structures. (b) In this example, the structure labeled "9" clearly does not belong in this cluster of fatty acyls. A possible explanation for the strange placement might be, that the "outlier"-structure has approximately the same myopic MCES distance to all other labeled compounds (18 or 19 except for structure "13", where the distance is 15); this is similar for the structure labeled "13" (albeit with a smaller distance: 10), which intuitively seems to be structurally much more closely related to the cluster. Numerous similar examples to those two cases can be found throughout the embedding.

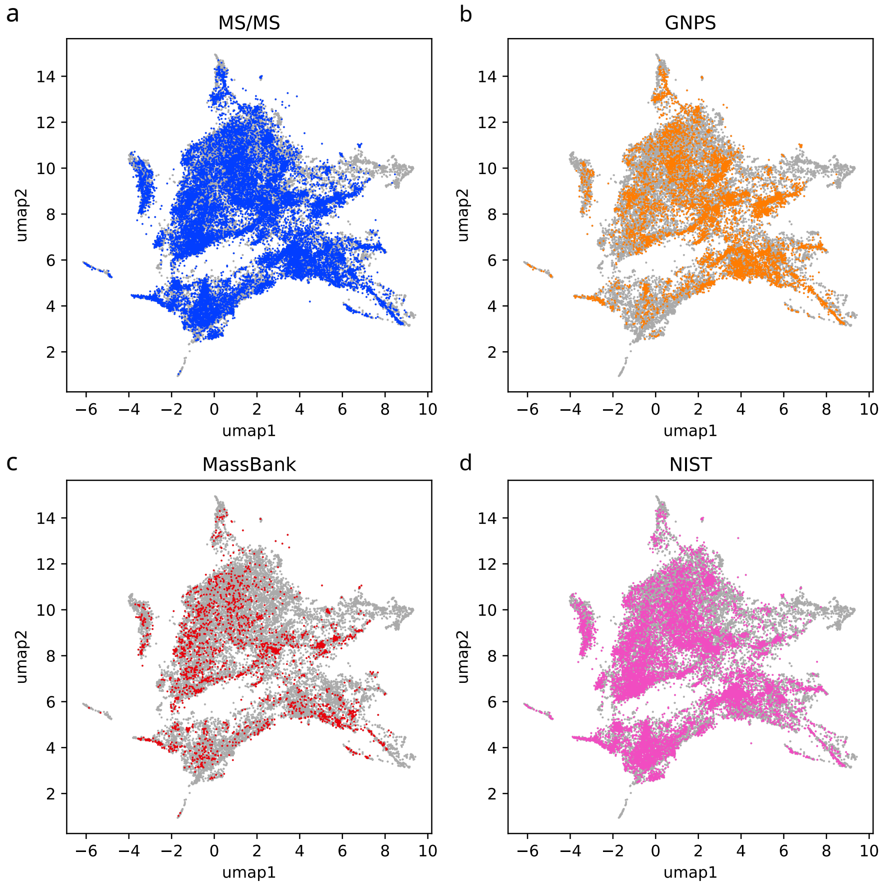

Supplementary Fig. 4. Maps of molecular structures in the MS/MS training dataset. Shown are both the total 18,848 molecular structures in the dataset (a) as well as those of the individual spectral libraries GNPS (b), MassBank (c), and NIST (d). A total of 36,944 molecular structures are shown in all plots combined. We stress that the assignment of molecular structures to the three libraries is not perfect: A molecular structure might be contained in more than one library but is displayed only for one. Hence, the plots *must not be overinterpreted to compare the coverage of the three libraries*. What we can learn from the three library plots is that each library covers the universe of biomolecular structures rather well. We use the same UMAP embedding as in Fig. 3. No molecular structures fall outside of this plot. Biomolecular structures are shown in light gray.

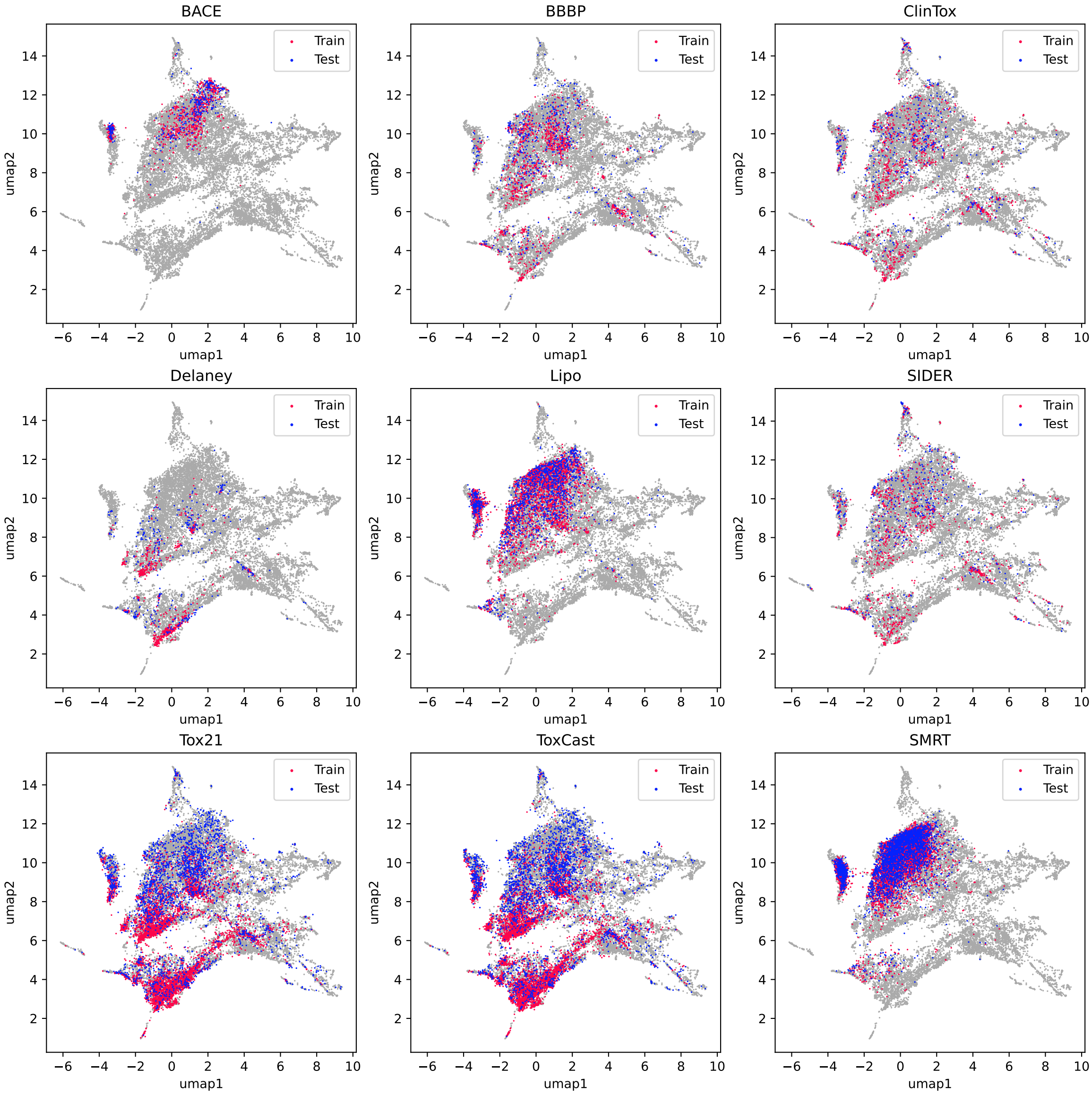

Supplementary Fig. 5. Map of nine scaffold splits. Molecular structures from the nine machine learning datasets from Fig. 5 were split according to Bemis-Murcko scaffolds. We used the `splits.ScaffoldSplitter.train_test_split` function from DeepChem (version 2.6.1) to generate a 70/30 split. For the datasets Delaney, SIDER, Tox21 and ToxCast, we observe that splits are clearly non-uniform. However, we do not observe a clear global separation between splits, for any datasets; instead, molecular structures of both train and test splits are spread across the whole covered structure space.

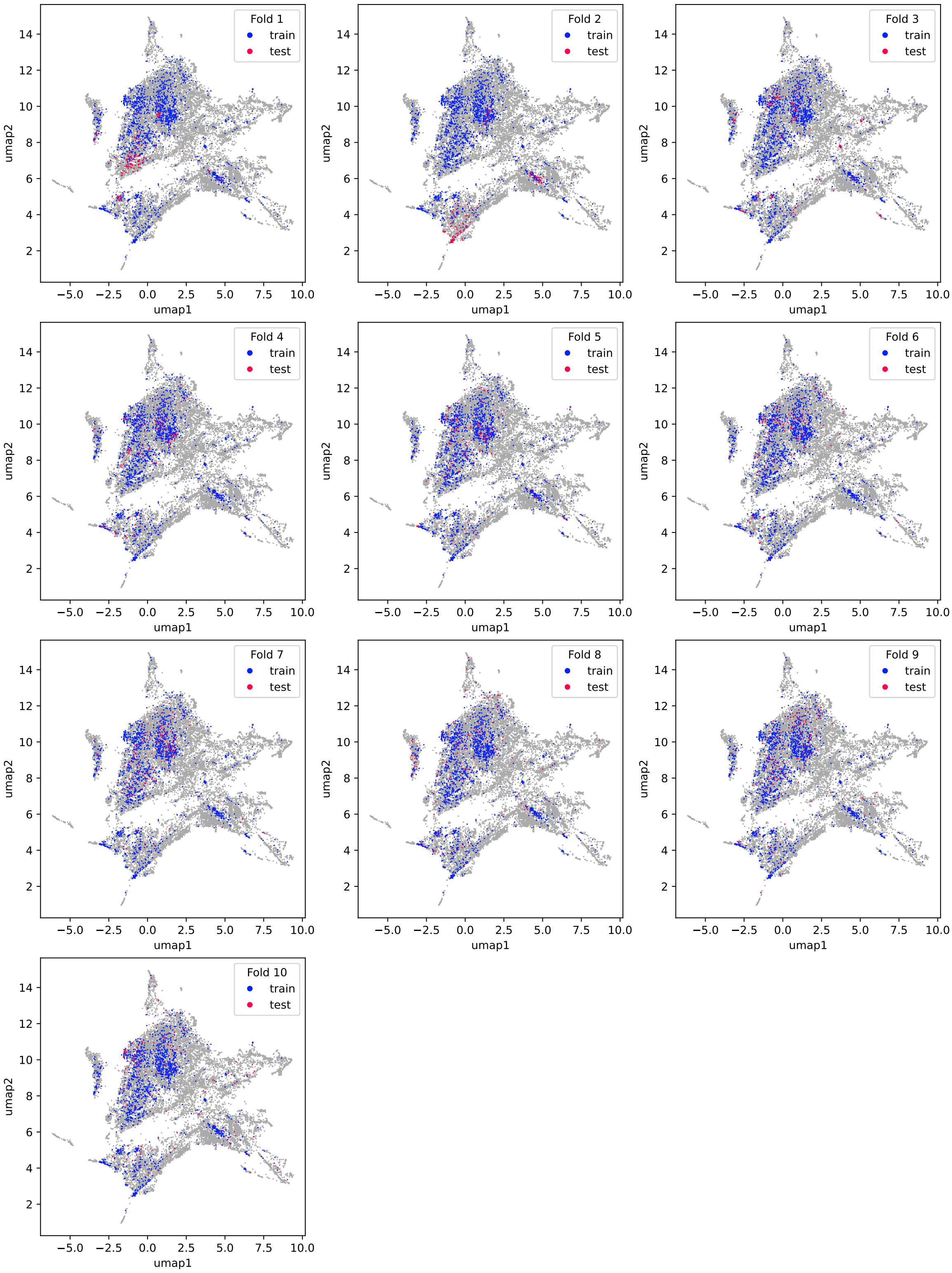

Supplementary Fig. 6. Map of the BBBP dataset with a 10-fold scaffold split. We generated a ten-fold scaffold split of the training dataset BBBP, compare to Supplementary Fig. 5. Here, we used the `splits.ScaffoldSplitter.k_fold_split` function from DeepChem. We observe that the split is clearly not uniform, in particular for the first two folds. Notably, this implementation of a cross-validation scaffold split sorts the scaffolds and, consequently, also splits based on size; this explains the increasing uniformity of the folds. We can observe small clusters where structurally similar molecular structures were sorted into the same fold.

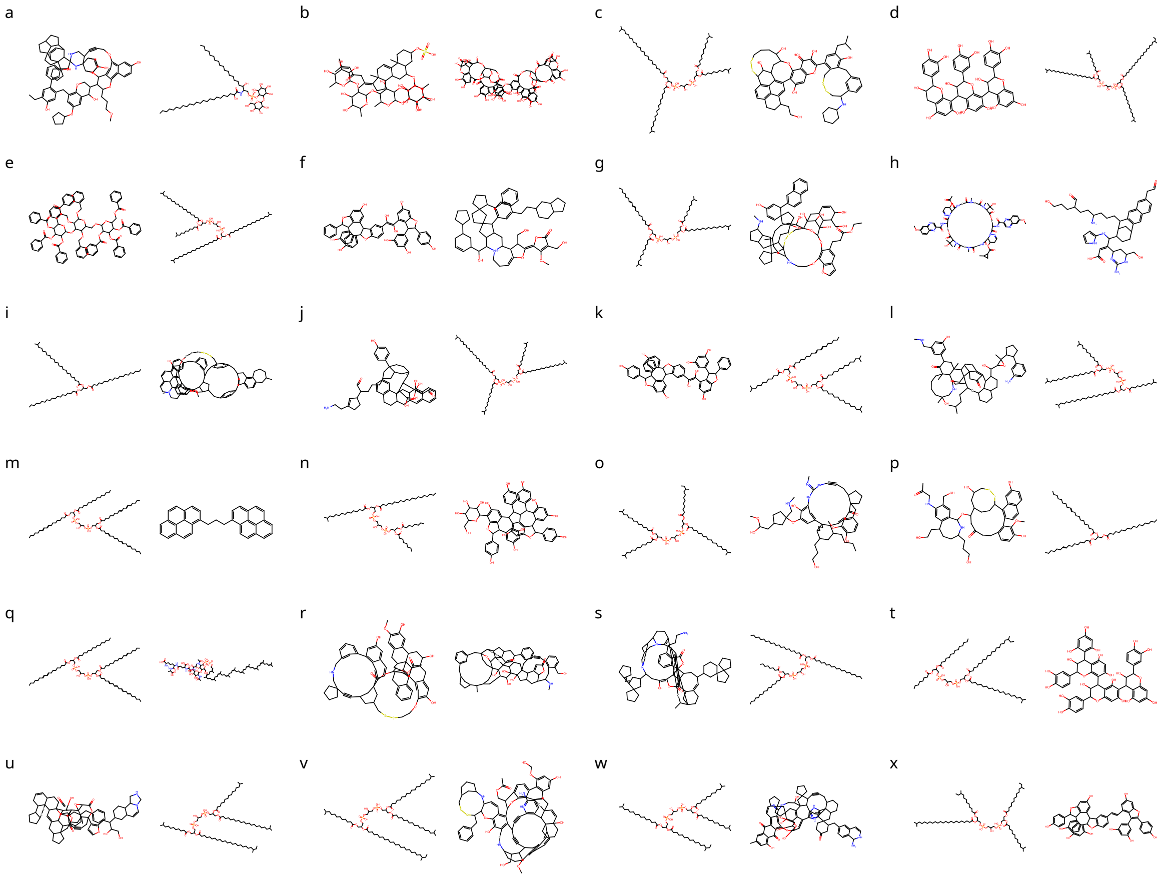

Supplementary Fig. 7. For 24 instances, exact MCES computations via Integer Linear Programming took more than four days. Forcing exact computation of the MCES distance for the subsampled 20,000 instances resulted in huge running times for a few instances, with 24 instances exceeding four days of wall clock time. Molecular structures of these instances are shown here (a–x). In all cases, structures are highly dissimilar and large, with myopic MCES distances (bounds only) ranging from 24.5 to 108. We may safely assume that for none of these instances, computing the exact MCES distance provides any useful information beyond that of the myopic MCES distance, which can be computed in fractions of a second.

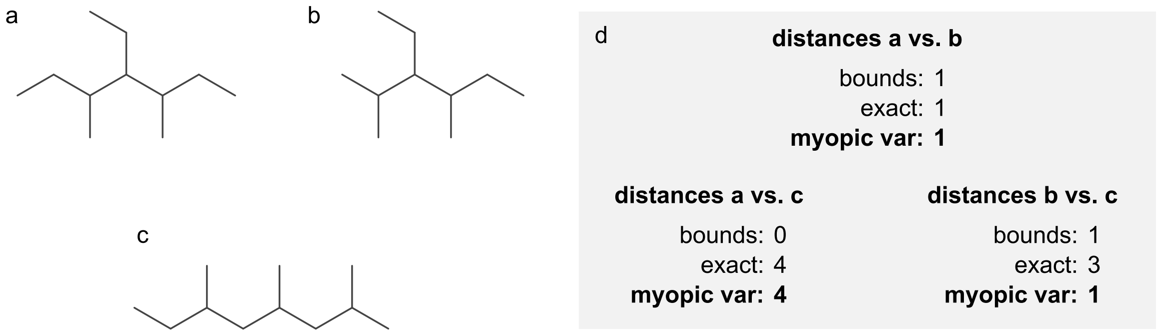

Supplementary Fig. 8. Double thresholding is needed to enforce the triangle inequality. Different from the myopic MCES distance used in the rest of the paper, we now consider a variant (“myopic var”) that does not employ double thresholding: We again first compute the MCES bounds, then decide whether the distance bound is below T. In this case, we compute the exact MCES distance. Different from the rest of the paper, we then use this exact MCES distance, *even if it is larger than* T. As shown in (d), the resulting distance violates the triangle inequality for molecular structures (a,b,c): For $T = 1$ the “myopic var” distance between a and c is 4, which is strictly larger than the sum of “myopic var” distances from a to b and from b to c. The problem is resolved for the myopic MCES distance as there, the distance between a and c is only $\min\{4, T\} = 1$.

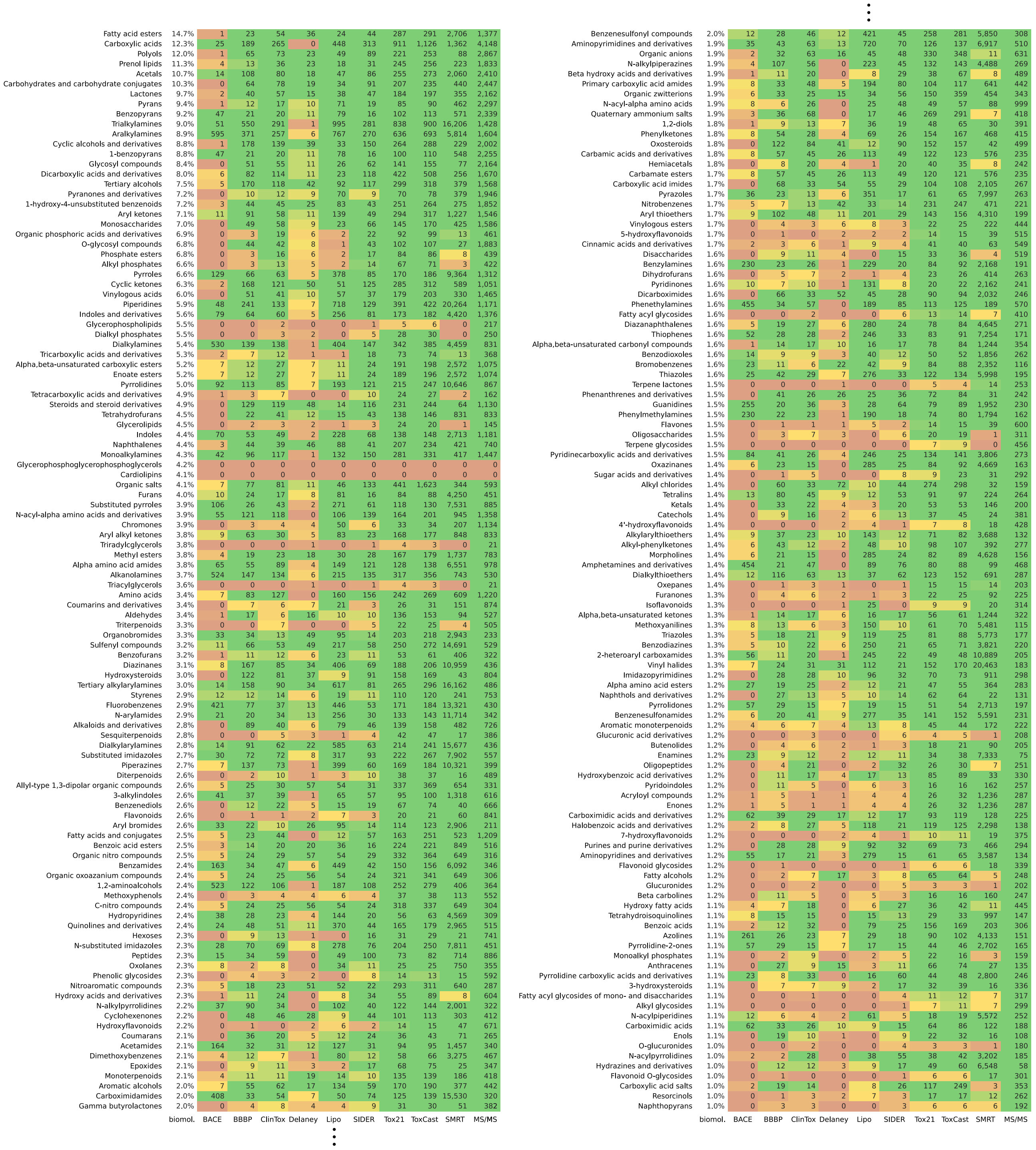

Supplementary Fig. 9. Compound class distribution. Similar to Fig. 7, the distribution of frequently occurring compound classes in all datasets described in this paper is shown. Here, classes occurring in at least 1 % of the biomolecular structures are displayed. Again, compound classes with at least 15 occurrences in all datasets are omitted; this applies to 95 compound classes.

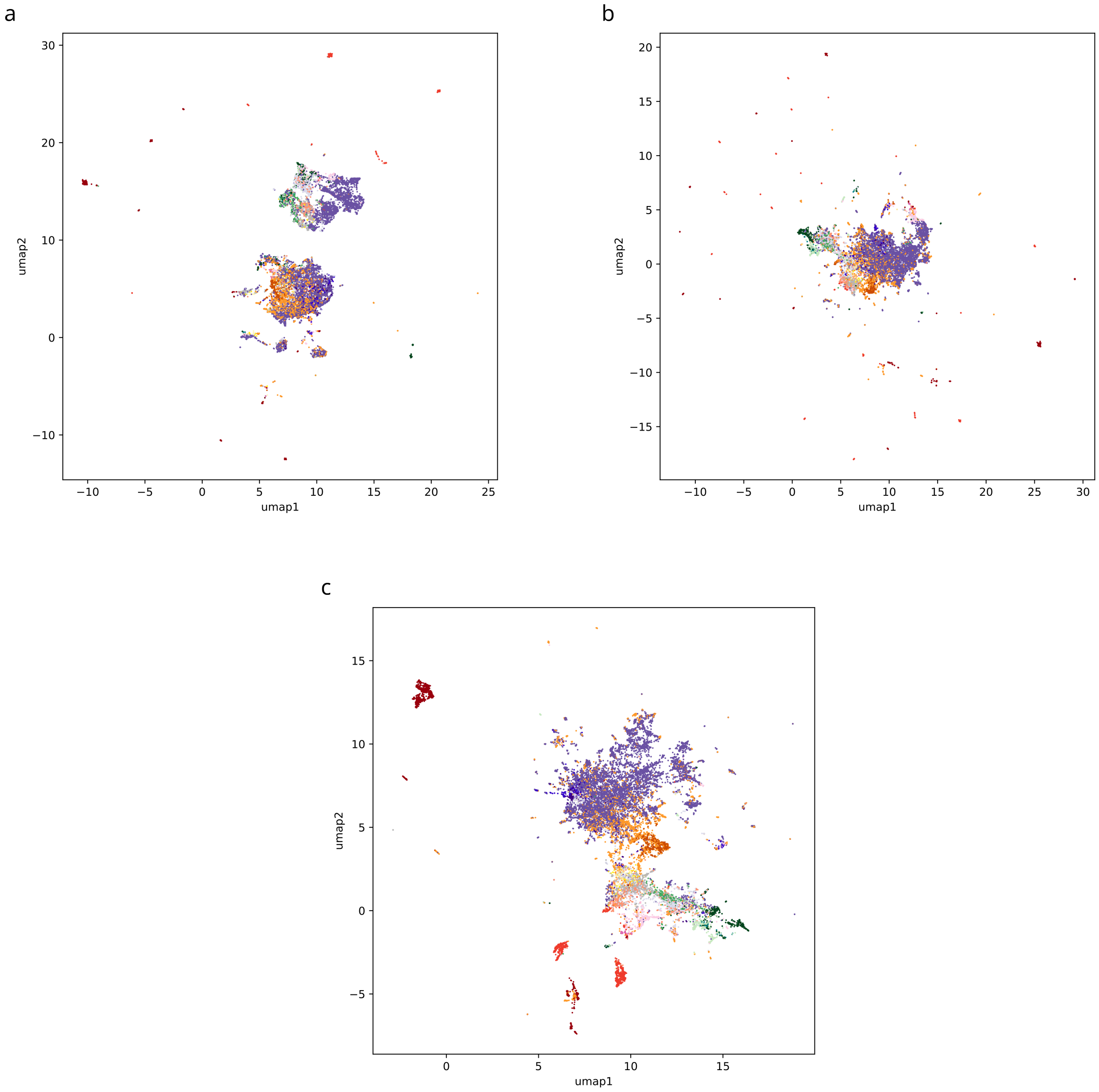

Supplementary Fig. 10. UMAP embedding using Tanimoto coefficients. We use and display all 19,994 biomolecular structures. Jaccard distances (one minus Tanimoto coefficients) were computed from MACCS fingerprints (a), ECFP4 (Morgan) fingerprints (b), and MAP4 fingerprints (c). The resulting distances are used to create corresponding UMAP embeddings. Compound classes are color-coded as in Fig. 1. Different from the MCES-based plots, for both types of fingerprints we observe numerous outlier clusters, as well as singleton outliers. Certain compound classes are separated in distant clusters. Compare to Fig. 1 where outliers were not removed.

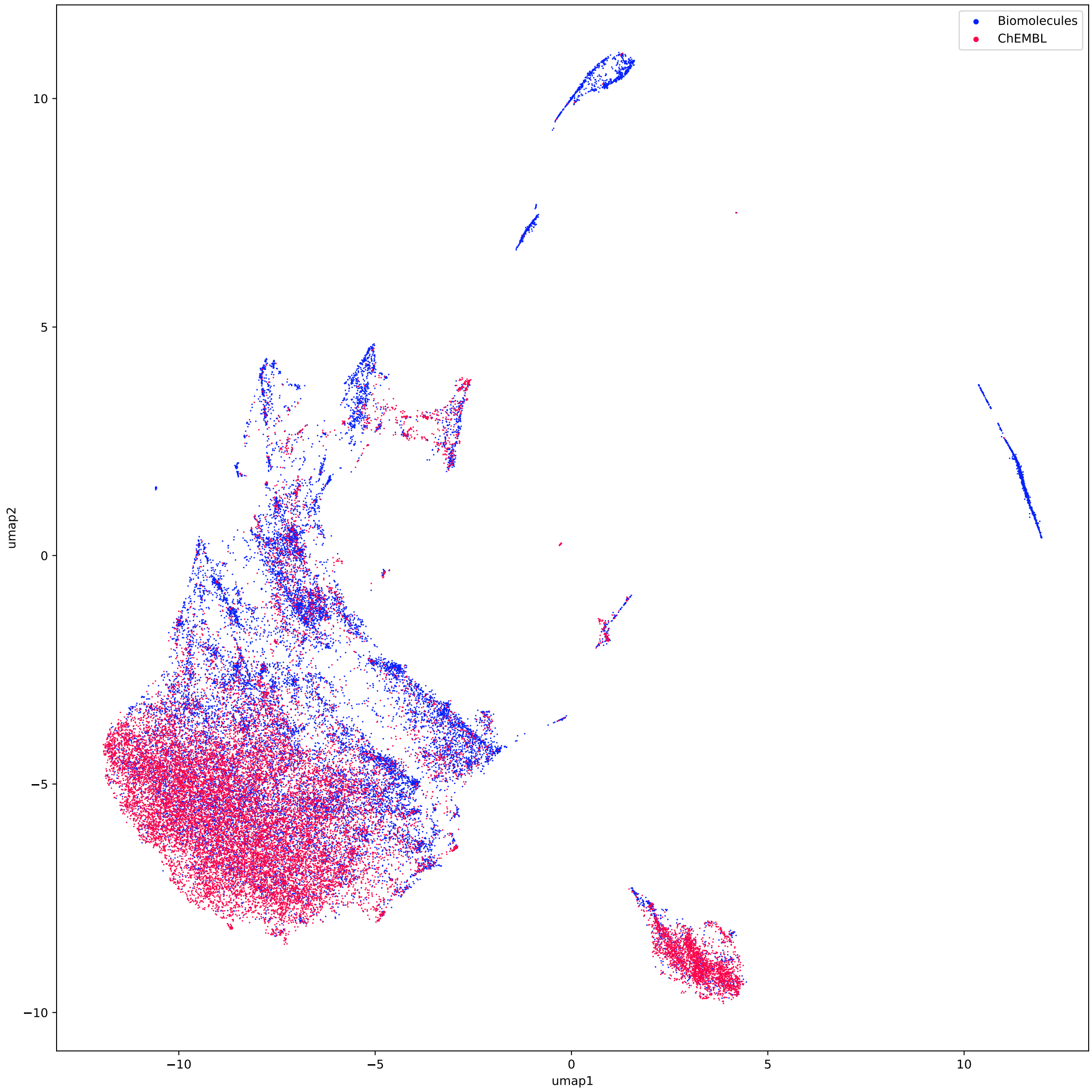

Supplementary Fig. 11. Joint UMAP embedding of ChEMBL and biomolecular structures. We uniformly subsample 20,000 structures from ChEMBL “small molecules”, downloaded on July 31st, 2024. For each structure that could not be standardized, not processed by rdkit, or consisted of single ions, another structure was drawn. UMAP embeddings were computed from the union of 20,000 ChEMBL structures and 19,9994 biomolecular structures. We clearly obsere different structure distributions for ChEMBL and the biomolecular structures. Compare to Fig. 1 where only biomolecular structures are shown, and to Supplementary Fig. 12 where ChEMBL structures are used as the background distribution.

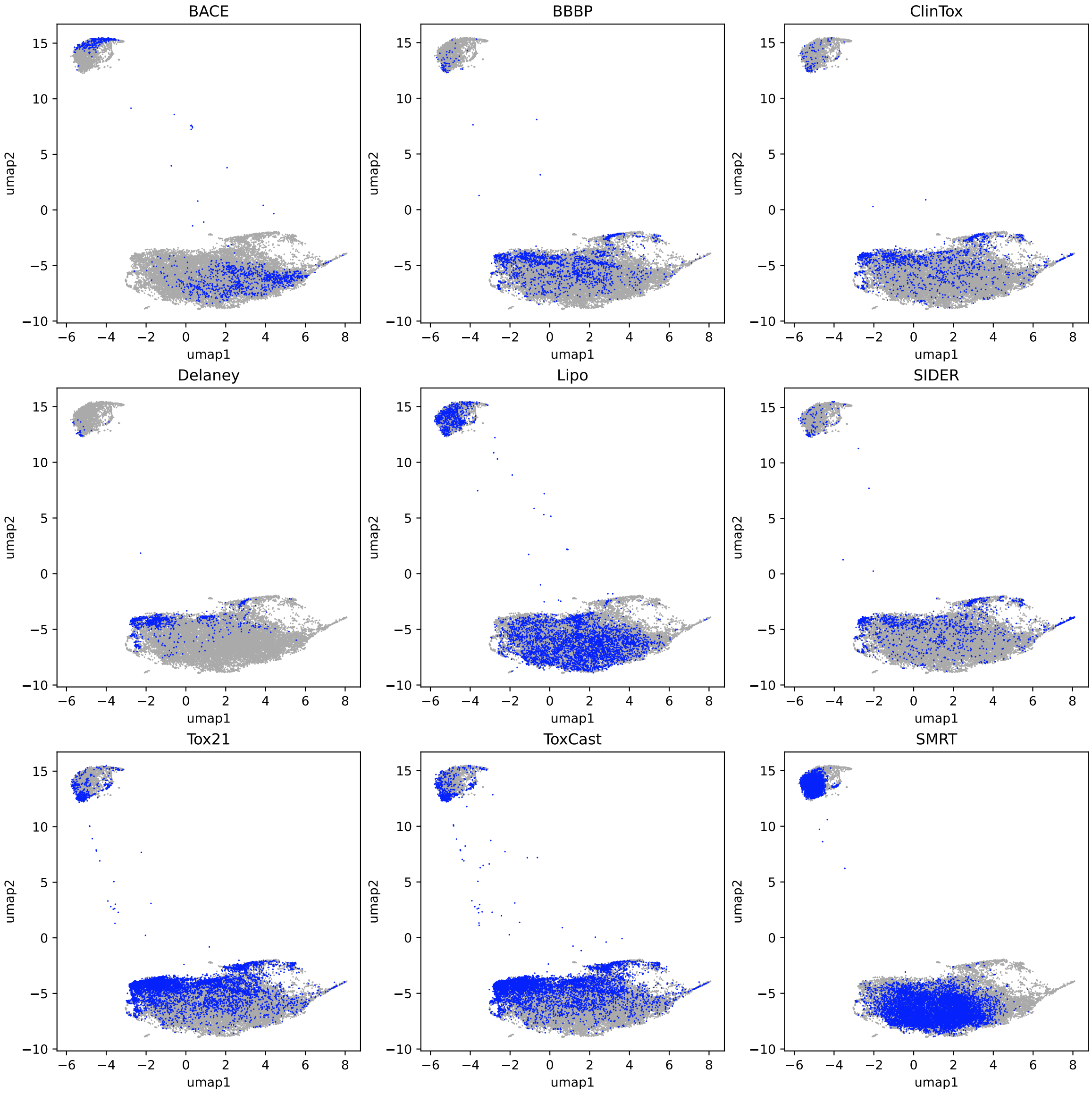

Supplementary Fig. 12. Maps of nine training datasets using ChEMBL as the background distribution. The 20,000 structures from ChEMBL (see Supplementary Fig. 11) were used to derive the UMAP embedding, and show in light gray. The same training datasets as in Fig. 5 are shown, see there for details. Notably, the background ChEMBL distribution is clearly separated between molecular structures that contain a sulfonyl group (upper-left cluster, 99.4% of molecular structures contain a sulfonyl group) and those that do not (rest, 0.5% of molecular structures contain a sulfonyl group). Similarly, 77.4% of molecular structures in the upper-left cluster contain a sulfonamide group, vs. 0.2% of the other molecular structures. Consistent with our analysis for biomolecular structures, most datasets show areas of missing coverage, with Tox21 and Toxcast providing the best coverage. Notably, the Lipo and SMRT datasets appear to be more uniform than in our previous analyses.

Supplementary Table 1. Structure databases that contribute to the set of biomolecular structures used herein. For each database the total number of molecular structures (total) and the number of structures that are absent of all preceding databases (additional) is shown. A subset of structures from PubChem (PC) which are annotated with categories of biological relevance is included.

Database	total	additional	Ref.	URL
KEGG	14,362	14,362	[82]	`https://www.kegg.jp`
ChEBI	56,219	42,621	[83]	`https://www.ebi.ac.uk/chebi`
HMDB	95,973	85,942	[84]	`https://www.hmdb.ca`
YMDB	1,864	135	[85]	`http://www.ymdb.ca`
PlantCyc	5,503	1,072	[86]	`https://plantcyc.org/`
MetaCyc	13,059	3,175	[87]	`https://metacyc.org`
KNApSAcK	42,168	31,967	[88]	`http://www.knapsackfamily.com/KNApSAcK/`
UNPD	161,976	112,057	[89]	`http://pkuxxj.pku.edu.cn/UNPD` [offline]
MaConDa	91	31	[90]	`https://www.maconda.bham.ac.uk/`
HSDB	4,319	1,080	[91]	`ftp://ftp.nlm.nih.gov/nlmdata/.hsdblease/`
Super Natural II	227,931	95,291	[92]	`http://bioinformatics.charite.de/supernatural`
COCONUT	380,169	104,404	[93]	`https://coconut.naturalproducts.net`
NORMAN suspect list	64,121	47,251	[94]	`https://www.norman-network.com/nds/SLE/`
MeSH-annotated PC	82,337	37,523	[66, 95]	`https://pubchem.ncbi.nlm.nih.gov`
PC “bio and metabolites”	138,753	30,500	[66]	`https://pubchem.ncbi.nlm.nih.gov/classification`
PC “drug”	7,649	651	[66]	`https://pubchem.ncbi.nlm.nih.gov/classification`
PC “safety and toxic”	148,962	109,792	[66]	`https://pubchem.ncbi.nlm.nih.gov/classification`
PC “food”	4,305	243	[66]	`https://pubchem.ncbi.nlm.nih.gov/classification`

Supplementary Table 2. Molecular structure dataset sizes. Number of molecular structures in the considered molecular structure training datasets. Some molecular structures failed the standardization via PubChem and were discarded, see column “After standardization”. Few molecular structures resulted in failed MCES computations and were also discarded, see column “After MCES computations”. For the *SMRT* dataset we uniformly subsampled 10,000 standardized molecular structures. The number “Total in plot” refers to the union of the molecular structures in the dataset and the 18,096 biomolecular structures.

Dataset	Number of compounds	After standardization	After MCES computations	Total in plot	ref.
BACE	1513	1194	1194	19,290	[41]
BBBP	2039	1662	1662	19,758	[42]
ClinTox	1478	1476	1475	19,571	[43]
Delaney	1128	1128	1127	19,223	[44]
Lipo	4200	4200	4200	22,296	[45]
SIDER	1427	1420	1389	19,485	[46, 47]
SMRT	80,037	10,000	10,000	28,096	[48]
ToxCast	8576	8517	8502	26,598	[49]
Tox21	7831	7783	7763	25,859	[50, 51]
MS/MS	18,848	18,848	18,848	36,944	[52, 53]
